# A high-sensitive room temperature gas sensor based on cobalt phthalocyanines and reduced graphene oxide nanohybrids for the ppb-levels of ammonia detection†

**DOI:** 10.1039/c9ra08065a

**Published:** 2019-11-18

**Authors:** ZhiJiang Guo, Bin Wang, Xiaolin Wang, Yong Li, Shijie Gai, Yiqun Wu, XiaoLi Cheng

**Affiliations:** Key Laboratory of Functional Inorganic Material Chemistry, Ministry of Education, School of Chemistry and Materials Science, Heilongjiang University Harbin 150080 P. R. China wangbin@hlju.edu.cn; School of Material and Chemical Engineering, Heilongjiang Institute of Technology Harbin 150050 P. R. China; Shanghai Institute of Optics and Fine Mechanics, Chinese Academy of Sciences Shanghai 201800 P. R. China

## Abstract

Highly sensitive gas sensing materials are of great importance for environmental pollution monitoring. In this study, four nanohybrid materials containing different phenoxyl substituents of cobalt phthalocyanines (tetra-β-carboxylphenoxylphthalocyanine cobalt (cpoPcCo), tetra-β-(4-carboxy-3-methoxyphenoxy)phthalocyanine cobalt (cmpoPcCo), tetra-β-phenoxylphthalocyanine cobalt (poPcCo), and tetra-β-(3-methoxyphenoxy)phthalocyanine cobalt (mpoPcCo)) and reduced graphene oxide (rGO) (RPcCo/rGO) were synthesized *via* non-covalent interactions as a high performance gas sensing materials for the ppb-level detection of ammonia (NH_3_). Various characterization techniques, including FT-IR, Raman, UV-vis, TGA, XPS and SEM, were used to confirm the structure, element information and morphology of the as-synthesized materials. The obtained materials were used in interdigital electrodes to fabricate the sensing device, and the gas sensing performance was investigated at room temperature. The obtained sensors exhibited excellent sensitivity, selectivity, good reproducibility and perfect response–concentration linearity towards NH_3_, which are mainly ascribed to the synergetic effects of RPcCo and rGO due to the specific surface area structure for NH_3_ diffusion, the abundant active sites to adsorb NH_3_, and excellent conductivity for efficient electron transport, particularly the effect of RPcCo. For example, the cpoPcCo/rGO-based sensor showed a higher and faster response for low concentration of NH_3_ (∼2.5 and 45 s for 100 ppb of NH_3_), a ppb level detection and superior stability over 60 days. Besides, the effect of different phenoxyl substituents of cobalt phthalocyanines on the sensing performance and the sensing mechanism for the sensitivity enhancement were discussed and confirmed by the first-principles density functional theory calculations and electrochemical impedance spectroscopy (EIS).

## Abstract 2

Highly sensitive gas sensing materials are of great importance for environmental pollution monitoring.

## Graphical abstract

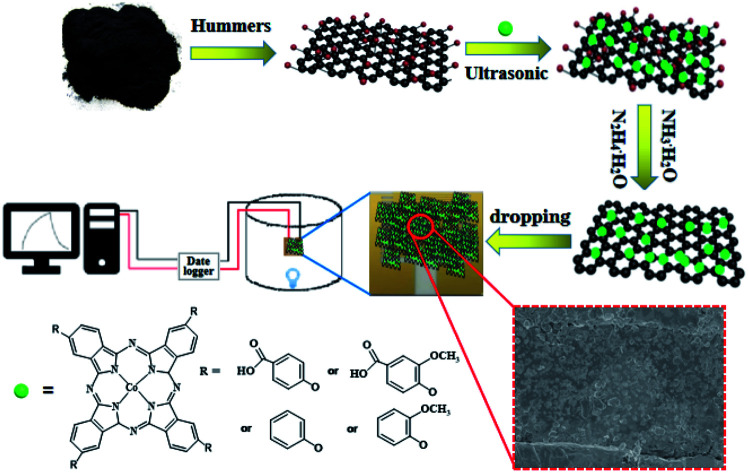

## 1. Introduction

In recent years, various toxic gases (such as NH_3_, CO_2_, SO_2_, and H_2_S) have been released due to rapid developments and unforeseen accidents in various industries. Hence, a technology to detect gaseous air pollutants and toxic gases has become critical.^1^ Among them, as a highly toxic gas, ammonia (NH_3_) usually comes from the factories, food industries and chemical industries. We know that it is extremely harmful to humans and the environment.^2^ When the concentration of NH_3_ exceeds 25 ppm, it will cause serious damage to human organs, such as the respiratory tract and eyes and also cause skin irritation.^3,4^ Therefore, it is very important to design and develop ammonia sensing materials with good selectivity, high sensitivity, fast response and recovery performance.

Recently, graphene nanosheets have attracted considerable attention of scientists, particularly graphene oxide (GO) and reduced graphene oxide (rGO). GO and rGO, as two-dimensional carbon materials, have a high specific surface area and unique properties, such as high thermal conductivity, good mechanical properties and rich functional groups (such as carboxyl, hydroxyl and epoxy) with excellent chemical stability; hence, they are considered to be promising gas-sensitive materials.^5,6^ In 2004, for the first time, Novoselov *et al.* found the possibility of inducing charge carriers to graphene by the adsorption of various gases including H_2_O, NH_3_ and NO_2_.^7^ Later, in 2007, graphene-based gas sensors were prepared for the adsorption of individual gas molecules for detection.^8^ Wehling *et al.* showed that graphene's peculiar density of states (DOS) was ideal for the “chemical sensor” application and explained the above study.^9^ Leenaerts *et al.* confirmed that NO_2_ and H_2_O acted as acceptors, and CO and NH_3_ acted as donors by analysing the interactions between different gas molecules and the GO surface using charge transfer analysis.^10^ These experimental and theoretical studies provided a reliable basis for graphene to be considered a good gas-sensitive material. Recently, GO and rGO-based gas sensors have been widely studied as gas sensing materials. Chen *et al.* showed that GO could detect a series of gas molecules at very low concentrations with detection limits of up to one million molecules.^11^ Li *et al.* reported that the response of pure rGO was 5.3% to 50 ppm of NH_3_.^12^ Zhou *et al.* reported that rGO prepared by spray coating showed a response value of less than 5% for 80 ppm NH_3_ detection.^13^ In summary, it can be seen that simple graphene has good electrical conductivity and high electromigration.^14,15^ However, simple GO and rGO show low sensitivity, poor selectivity and slow recovery capability, which does not meet the standard requirements of practical application.^16^

Functional modification is a very reliable way to improve the gas sensing performance of graphite oxide and reduced graphene oxide. For example, doping some heteroatoms in graphene and combining with other gas sensing materials represent a new research method for improving the sensitivity of the hybrid.^17^ An ammonia gas sensor based on polyaniline with N-doped graphene quantum dots was prepared, and showed a response value of 110.92 for 1500 ppm NH_3_.^18^ Karaduman *et al.* reported that the rGO hybrids showed enhanced NH_3_-sensing properties by the modification of Ag, Au and Pt nanoparticles at room temperature.^19^ The rGO-TiO_2_ hybrid-based sensor has shown excellent sensing properties by improving the morphology of rGO sheets due to the introduction of synthetic TiO_2_.^20^ However, it is still a big challenge for GO and rGO hybrids to obtain excellent sensitivity, selectivity, and good stability.

It is well-known that metal phthalocyanines (MPcs) are also good gas sensitive materials due to their unique conjugated 18π-electron structure.^21^ There are 16 hydrogen atoms around the phthalocyanine ring, which can be substituted by other groups, such as alkoxy group, carboxyl group, amino group and sulfonic acid group^22–24^ which provides an opportunity to improve and optimize the NH_3_-sensing performance of rGO by using substituted MPcs as dopants. In addition, there is a hole in the ring that can accommodate a variety of different metal elements to form different kinds of metal phthalocyanines. This structure is conducive to further optimize the NH_3_-sensing performance due to highly active central metal atoms for selective NH_3_ adsorption. More importantly, the unique planar 18π-electron-conjugated structure of MPcs allows them to be closely coupled with the rGO by π–π interactions.^25^ On the one hand, the MPcs provide more gas sensing active sites for selective NH_3_ adsorption. On the other hand, rGO provides fast and efficient charge transfer.^26,27^ Apparently, the NH_3_-sensing performance of MPc/rGO hybrids will be greatly improved.^28^ However, MPcs and rGO hybrids have rarely been reported as gas sensing materials.

**Scheme 1:**
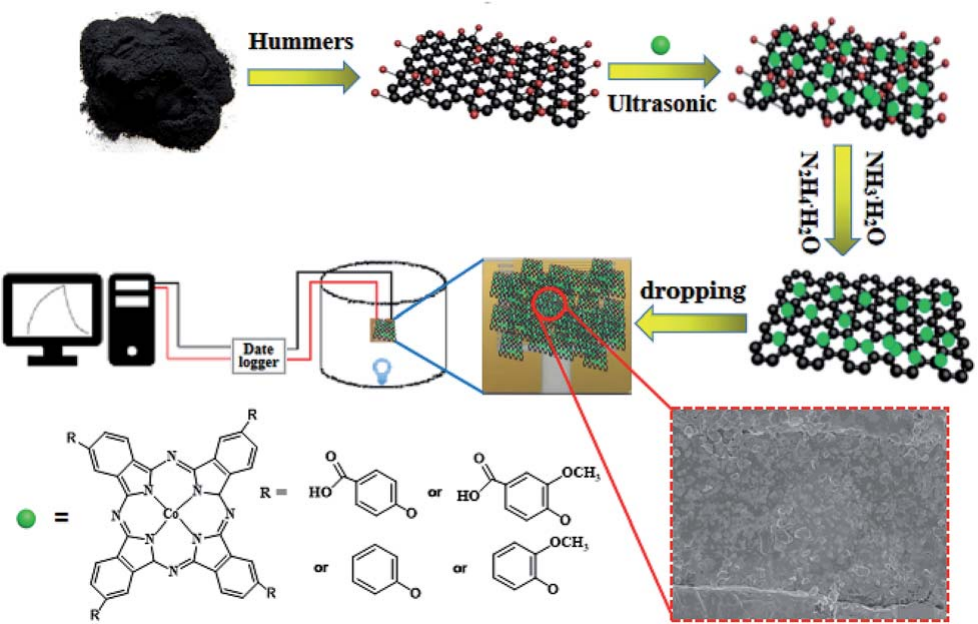
Synthesis and processing schematic of RPcCo/rGO hybrids and gas sensor device.

Based on the above consideration, herein, four nanohybrid materials containing different phenoxyl substituents of cobalt phthalocyanines (tetra-β-carboxylphenoxylphthalocyanine cobalt (cpoPcCo), tetra-β-(4-carboxy-3-methoxyphenoxy)phthalocyanine cobalt (cmpoPcCo), tetra-β-phenoxylphthalocyanine cobalt (poPcCo), and tetra-β-(3-methoxyphenoxy)phthalocyanine cobalt (mpoPcCo)) coupled with reduced graphene oxide (RPcCo/rGO) were successfully prepared (see Scheme 1). The effect of the substituents of cobalt phthalocyanines on gas sensing performance was investigated. The presence of different substituents on the phthalocyanine greatly affects the gas sensitivity of the RPcCo/rGO hybrids. When the electron-withdrawing carboxyl group is present on the phenoxyl substituents of the phthalocyanine ring, it increases the hole sites of the phthalocyanine and provides a lot of active sites for NH_3_ adsorption. Therefore, the cpoPcCo/rGO hybrid exhibited a high gas response of 42.4% for 100 ppm of NH_3_ with a reversible recovery time of 120 s and a very low detection limit of 3.7 ppb. Furthermore, the selectivity, stability and the NH_3_-sensing mechanism have also been studied in detail.

## 2. Experimental and calculation details

### 2.1 Materials and reagents

Flake graphite was purchased from Shenzhen Nanotech Port Co. Ltd. 4-Nitrophthalonitrile (99% purity), phenol (98% purity), methoxyphenol (98% purity), *p*-hydroxybenzoic acid (99.5% purity), sodium 4-hydroxy-3-methoxybenzoate (>98% purity) and DBU (98% purity) were purchased from Sigma-Aldrich Co. LLC. Ultrapure water (resistivity 18.2 MΩ cm) was obtained from a Milli-Q Water System (Millipore Corp., Bedford, MA, USA) and was used throughout the experiments. Graphene oxide (GO) was fabricated using the modified Hummers' method from graphite powder, which was described in our former reports.^29^ Tetra-β-carboxyphenoxyphthalocyanine cobalt(ii) (cpoPcCo) was synthesized by the universal template reaction of 4-(4-carboxyphenoxy) phthalonitrile with anhydrous cobalt(ii) chloride in the presence of 1,8-diazabicyclo[5,4,0]undec-7-ene (DBU). The other cobalt phthalocyanine(ii) is compounded similarly and only 4-(4-carboxyphenoxy)phthalonitrile is replaced (Scheme S1, see Experiment details in ESI†). All other reagents were of analytical grade and utilized without further purification.

### 2.2 Preparation of PcCo/rGO hybrids

Four RPcCo/rGO hybrids, poPcCo/rGO, mpoPcCo/rGO, cpoPcCo/rGO, cmpoPcCo/rGO, were prepared by the uniform method: GO (0.100 g) was sonicated in *N*,*N*-dimethylformamide (DMF) solution (30 mL) at room temperature for 4 h. The corresponding PcCo (0.200 g) was dissolved in DMF solution (10 mL), and then the PcCo solution was added to GO solution dropwise. The ensuing mixture was sonicated for further 48 h at room temperature. Then, hydrazine hydrate (0.6 mL) and ammonia (4 mL) were added and stirred at 100 °C for 24 h under a nitrogen atmosphere. After cooling, the resultant solution was filtered through a 0.20 μm Millipore, and washed successively by DMF, ethanol and acetone until the filtrate was colourless. Subsequently, the resultant product was dried in a vacuum at 60 °C for 5 h. In addition, rGO was prepared using the same procedure as used for RPcCo/rGO hybrids but without the addition of RPcCo, and RPcCo covalently linked rGO (RPcCo-rGO) was also prepared using a method reported in literature.^30^

### 2.3 Sensor assembling and sensing measurements

An entire description of the gold electrodes and the gas sensor testing device was illustrated in our previous research, (the detailed structure parameters for gas sensors see Experiment details in ESI†).^29,31^

To prepare gas sensors composed of RPcCo/rGO hybrids, the as-prepared RPcCo/rGO hybrids were dispersed in ethanol to attain a uniform suspension of 1.0 mg mL^−1^ ultrasonication for 2 h, and then about 50 μL of dispersion was dropped on the interdigitated electrodes using a microsyringe. After the evaporation of the solvent, any residue of the solvent left was thoroughly removed by drying the sensor devices in a vacuum oven for 2 h at 80 °C. By contrast, the gas sensors of rGO and RPcCo-rGO were also fabricated by the similar procedures.

A classic sensing test cycle composed of three sequential steps to speed up the recovery of the sensor, a mini ultraviolet (UV) lamp (10 V/3 W, 254 nm) was used to removal of the gas. First, airflow was introduced into the sensing test chamber to obtain a baseline, and then the chamber was vacuumed by the pump. Later, a standard gas was injected to the chamber by a syringe, and then RPcCo/rGO hybrid sensor was placed in testing gas to register sensor signals. Finally, the sensor was recovered in the air under UV light. All measurements were performed at 29 °C ± 0.5 °C with a relative humidity of 55% ± 5%. In this study, sensitivity (*S*) is defined by the relative resistance change, as follows:1
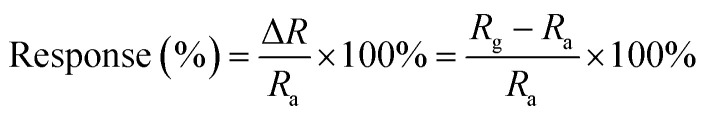
where *R*_a_ is the sensor resistance in the initial airflow, which was used as the background and *R*_g_ is the sensor resistance after being exposed to a certain concentration of target gas. Response and recovery times are defined as the time needed for 90% of total resistance change on exposure to the target gas and air, respectively. The sensor responses to different RH were measured at 29 °C ± 0.5 °C, and a certain RH level was achieved by mixing dry air and water vapour.^23^

### 2.4 Characterization

UV/vis absorption spectra were recorded using a Lambda 35 UV/vis spectrometer (PerkinElmer, USA). FT-IR spectra were recorded on a Nicolet FT-IR NEXUS spectrometer (Thermo Scientific). The Raman spectra were obtained using a Raman spectrophotometer (HR800, HORIBA JobinYvon Company) exploited by a laser with a 457.9 nm wavelength. Scanning electron microscopy (SEM) images were recorded using a Hitachi S-4800 field emission scanning electron microscope, operating at 15 kV. Transmission electron microscope (TEM) images were recorded on a JEM-3010 electron microscope (JEOL, Japan) at an acceleration voltage of 300 kV. X-ray photoelectron spectroscopy (XPS) measurements were performed using AXIS UL TRA DLD. Thermogravimetric (TG) analysis was performed on TA Q600 under a stream of nitrogen at a heating rate of 10 °C min^−1^.

### 2.5 Calculation details

DFT calculations were performed for the adsorption of NH_3_ on RPcCos, using long-range corrected functional of CAM-B3LYP with a set of hybrid basis sets (LanL2DZ for metals and 6-31G(d) for H, C, and N).^32^ Charge analyses were performed with the NBO (natural bond orbital) method.^33^ The Gaussian 09 quantum chemical package was employed for all calculations in the present study.^34^

## 3. Results and discussion

### 3.1 Characterization of RPcCo/rGO hybrids

**Fig. 1:**
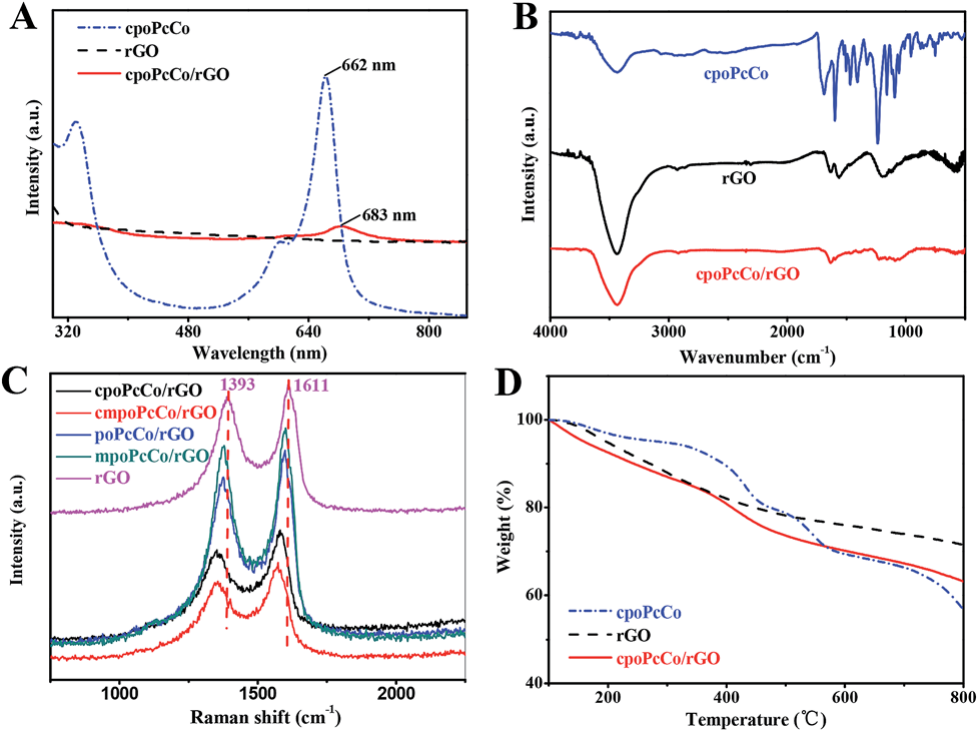
(A) UV-vis spectra of rGO, cpoPcCo and cpoPcCo/rGO hybrids in DMF; (B) FT-IR spectra of rGO, cpoPcCo and cpoPcCo/rGO hybrids; (C) Raman spectra of rGO, RPcCo/rGO hybrids obtained at *λ*_exc_ = 457.9 nm; (D) TG profiles of rGO, cpoPcCo and cpoPcCo/rGO hybrid.

The absorbance spectrum represents an important part of the UV-vis spectrum of phthalocyanine complexes. Fig. 1A and S2† show the UV-vis spectra of rGO, RPcCos and RPcCo/rGO in DMF. cpoPcCo, cmpoPcCo, poPcCo and mpoPcCo display maximum peaks at 662, 665, 664 and 668 nm, respectively, which are the characteristic absorptions of phthalocyanine in the Q-band region at around 650–700 nm, attributed to the π–π* transitions from the HOMO (highest occupied molecular orbital) to the LUMO (lowest unoccupied molecular orbital) of the Pc^2−^ ring.^35^ Because of the π–π interactions, RPcCo could spontaneously assemble with rGO, generating RPcCo/rGO hybrids. As shown in Fig. 1A and S2,† the characteristic absorption in the Q-band of RPcCo is observed in the spectra of RPcCo/rGO hybrid. Moreover, the RPcCo/rGO hybrids were further confirmed by the disappearance of the characteristic absorption of RPcCo in the absorbance spectrum of rGO. In addition, compared with the Q-band absorption of RPcCo, the Q-band absorption of the RPcCo/rGO hybrids red-shifted and broadened due to the reduction in the HOMO–LUMO energy gap of the phthalocyanine ring by π–π interactions between the Pc and rGO.^36,37^ Moreover, the Q band absorption of the substituted MPcs red-shifted in comparison with that of unsubstituted MPcs, because of the reduction in the HOMO–LUMO energy gap of the Pc ring by the introduction of the substituents.^35^ In the case of the presence of different substituents on the Pc ring, these band absorption peaks are different due to the electron-withdrawing and donating properties of the substituents. The Q-band of cmpoPcCo red-shifted (3 nm) in comparison with that of cpoPcCo due to the electron-donating properties of the methoxy substituent and was blue-shifted (3 nm) in comparison with that of mpoPcCo due to the electron-withdrawing properties of the carboxyl substituent. Because of the existence of lone pair electrons in NH_3_, the response interactions between NH_3_ and carboxyl-substituted PcCo could be increased due to the electron-withdrawing properties of the carboxyl substituent on the Pc ring, thus helping to increase its overall ammonia sensitivity.

The successful combination of RPcCos to rGO to give RPcCo/rGO hybrids was verified by FTIR. Fig. 1B and S3† show the FT-IR spectra of RPcCo, rGO and RPcCo/rGO hybrids. The spectrum of the cpoPcCo/rGO hybrid (Fig. 1B) shows the characteristic vibrations at 1636 cm^−1^ and 3440 cm^−1^ for *ν*C=C and *ν*O–H of rGO, respectively, while the characteristic fingerprints of cpoPcCo bands were also present at 1000–1600 cm^−1^, thus providing a significant support for the formation of cpoPcCo/rGO hybrid.^30,38,39^ The cmpoPcCo/rGO, poPcCo/rGO, and mpoPcCo/rGO hybrids (Fig. S3†) show very similar FT-IR spectra with the cpoPcCo/rGO hybrid. A significant π-electron interaction between the donor PcCo and the acceptor rGO basal plane was further confirmed by Raman spectroscopy. As shown in Fig. 1C, there are two characteristic bands at about 1393 cm^−1^ (D-band) and 1610 cm^−1^ (G-band) in the Raman spectrum of rGO. Compared with those of rGO, both the D- and G-bands of the RPcCo/rGO hybrids were shifted to lower wavenumbers due to the π–π conjugated interactions between RPcCo and rGO, the increase in charge carrier abundance provided by the PcCo molecules on the graphene-based surface, which ultimately increases the Fermi level,^40^ well-consistent with the ZnPc-GO hybrid.^41^ In addition, the order of the blueshift of the PcCo/rGO complex from small to large is cpoPcCo/rGO, cmpoPcCo/rGO, poPcCo/rGO and mpoPcCo/rGO. The blueshift increased with the increase in the electron-withdrawing properties of the substituent on the Pc ring. This has a great relationship with the charge transfer rate between PcCo and rGO and the effect of different substituents on the metal phthalocyanine on the complex.

The amount of cpoPcCo assembled on rGO was evaluated by TG analysis under N(g) atmosphere, as shown in Fig. 1D. The weight loss in rGO and cpoPcCo was about 28.48% and 43.20% in the range of 100–800 °C, respectively, due to the decarboxylation of the rGO surface oxide species, destruction of residual amorphous carbon and decomposition of cpoPcCo. In the same temperature range, the cpoPcCo/rGO hybrid revealed a 36.85% weight loss. From the weight loss of rGO, the corrected weight loss caused by cpoPcCo was estimated to be 8.37%. In fact, considering the weight loss of cpoPcCo and rGO, the amount of cpoPcCo adsorbed on the surface of rGO can be calculated as an actual ratio of 19.37 (8.37%/43.20%). Simultaneously, TG analysis images of cpoPcCo/rGO, poPcCo/rGO and mpoPcCo/rGO complexes were obtained, as shown in Fig. S4.† The amount of cmpoPcCo, poPcCo and mpoPcCo assembled on rGO were 16.02%, 21.04% and 18.61%, respectively. These results also demonstrate the successful preparation of the RPcCo/rGO hybrids. It can also be seen that the amount of RPcCo complex adsorbed on rGO was very similar.

**Fig. 2:**
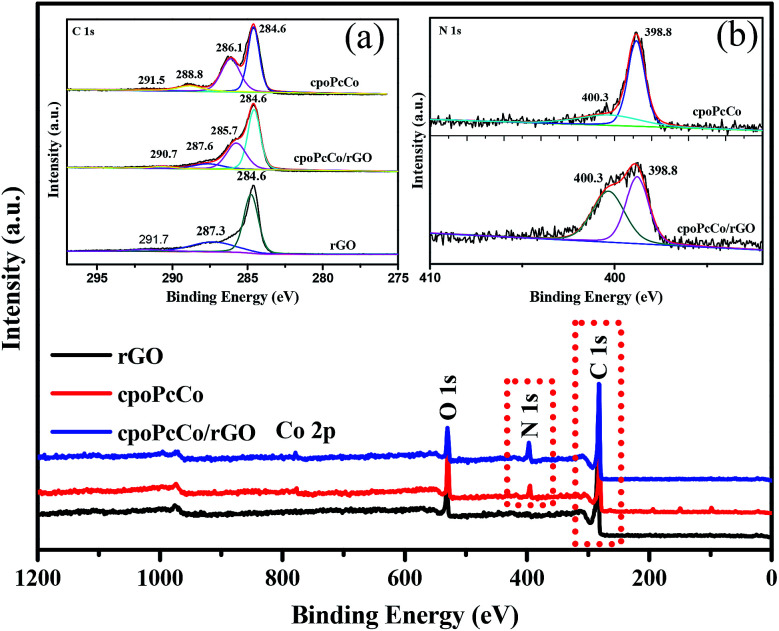
XPS full survey spectra of rGO, cpoPcCo and cpoPcCo/rGO hybrid; the inset images of (a) and (b) are the high-resolution XPS spectrum of C 1s and N 1s of the rGO, cpoPcCo and cpoPcCo/rGO hybrids.

XPS was also conducted to investigate the as-prepared surface chemical composition of RPcCo/rGO hybrids. As can be seen in Fig. 2, the distinct N 1s and Co 2p peaks emerge in the survey spectra of cpoPcCo/rGO hybrids compared to that of rGO. In the high-resolution C 1s XPS spectrum of cpoPcCo/rGO hybrid (the inset image of (a) in Fig. 2), the peak for cpoPcCo, rGO situated 291.5 eV, 291.7 (C=O) shifts to 290.7 eV, the peak for cpoPcCo, rGO situated 288.8 eV, 287.3 (C–OH) shifts to 287.6 eV. Compared with the C 1s XPS spectrum of rGO, the new peak situated 286.1 eV (C–N) was derived form cpoPcCo, which appears and shifts to 285.7 eV.^30,39^ In addition, the peaks at 398.7 eV (aza bridge nitrogen) and 400.1 eV (pyrrole nitrogen) can be observed in the high-resolution N 1s XPS spectrum of the cpoPcCo/rGO hybrid (the inset image of (b) in Fig. 2), which was also derived form cpoPcCo. The XPS spectrum of the cpoPcCo/rGO hybrid contains all the peaks, corresponding to the cpoPcCo and rGO. Similar results can be observed in XPS spectra of others the RPcCo/rGO hybrids (Fig. S5–S7†). All of this evidence further suggests that RPcCo is successfully combined with the rGO.

### 3.2 Gas sensing properties

**Fig. 3:**
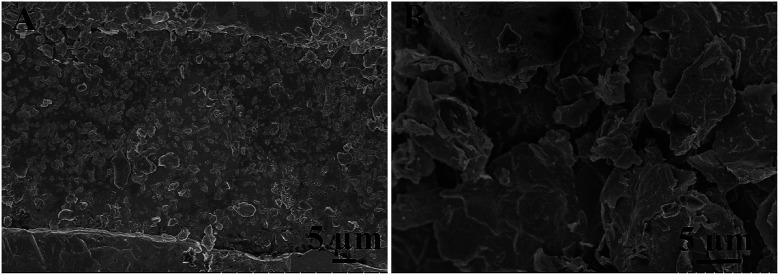
SEM images of cpoPcCo/rGO hybrids on the interdigital electrode, respectively.

The surface morphology of the RPcCo/rGO hybrid prepared on the interdigitated electrode was investigated by SEM, as shown in Fig. 3 and S8.† It can be clearly seen that RPcCo/rGO hybrids display a few-layered flake like shape with a rounded morphology. Fig. 3 shows that cpoPcCo/rGO sheets are evenly, loosely distributed between the two-finger of the interdigitated electrode, which provides continuous conducting pathways for the transportation of electrons, the permeable channels for the diffusion of gas molecules, and more advantages on the active sites, giving rise to the high sensing performance.^42,43^

**Fig. 4:**
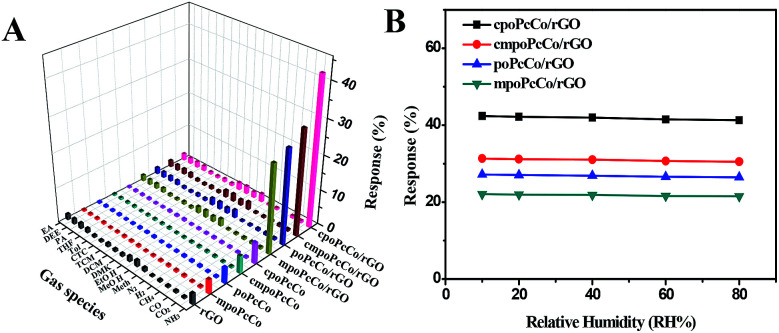
(A) Response of RPcCos, rGO and RPcCo/rGO hybrid-based sensors to 100 ppm of NH_3_ and 5000 ppm of others various gas, respectively. (B) Response of RPcCo/rGO hybrid-based sensors to 100 ppm NH_3_ in different relative humidity at 29 °C.

The gas sensing properties of RPcCos-based, rGO-based and RPcCo/rGO hybrid-based sensors deposited onto the interdigitated electrode were investigated by using ammonia as the target gas. The selectivity is an important characteristic for the application of sensors at room temperature. The response of nine sensors to NH_3_ gas with a concentration of 100 ppm and 17 different gases with a concentration of 5000 ppm was investigated, and the results are shown in Fig. 4A. The RPcCo/rGO hybrid-based sensors exhibited the highest response to NH_3_, particularly the cpoPcCo/rGO hybrid to 100 ppm of NH_3_ (42.4). The RPcCo/rGO hybrids showed the excellent selectivity toward NH_3_ as opposed to other gases. As for other sensors, the PcCos was not high enough, but showed a relatively good selectivity to NH_3_. On the contrary, the rGO sensor showed a poor response and selectivity to NH_3_, which was consistent with the reported literature (Fig. 4A and S9†).^44^ The results indicated that the introduction of RPcCo could significantly enhance the response and selectivity to NH_3_ due to strong interactions between the central metal of RPcCo and NH_3_.^45^ The effect of humidity on sensors is also an important factor for the application of sensors at room temperature. So, the response of the RPcCo/rGO hybrid sensors to 100 ppm of NH_3_ was tested under different relative humidities at 29 °C. As shown in Fig. 4B, the response of RPcCo/rGO hybrid sensors to 100 ppm NH_3_ was almost unchanged in different RH, which indicated that the as-prepared material possessed strong stability in a practical environment. Based on the results discussed above, the RPcCo/rGO hybrid sensors display superior selectivity and low effect of RH in its sensing response. Therefore, the RPcCo/rGO hybrids are promising candidates for use as a NH_3_ sensing material.

**Fig. 5:**
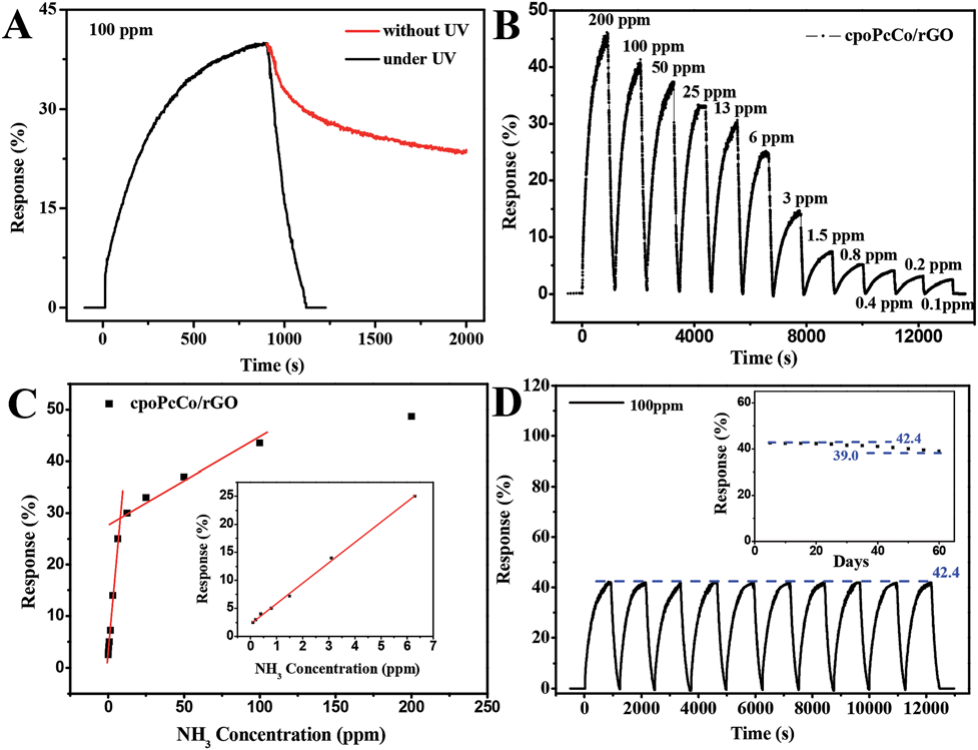
(A) Response of the cpoPcCo/rGO hybrid sensor exposure to 100 ppm NH_3_ and recovery under UV light or without UV light; (B) resistance of the cpoPcCo/rGO hybrid sensor upon exposure to varying concentrations of NH_3_; (C) relationship of the response of the cpoPcCo/rGO hybrid sensor to the concentration NH_3_; (D) ten sensing cycles of the cpoPcCo/rGO hybrid sensor to 100 ppm NH_3_ (inset: the reproducibility characteristics of the cpoPcCo/rGO hybrid sensor to 100 ppm NH_3_ within 60 days) at 29 °C.

To further investigate the NH_3_ sensing properties of RPcCo/rGO hybrid sensors, the signal resistor of the four RPcCo/rGO hybrid-based sensors were exposed to different concentrations of NH_3_, the signal resistor was continuously recorded, and then recovered under UV light at room temperature. As shown in Fig. 5A, the recovery time of cpoPcCo/rGO was about 180 s with a concentration of 100 ppm NH_3_ under UV light, but the recovery property was very poor without UV light. It was found that the recovery of the cpoPcCo/rGO hybrid strongly depends on UV assistance, which was consistent with other RPcCo/rGO hybrids in Fig. S10–S12.† Under UV irradiation, light-generating electrons can react with O_2_ in the air to produce adsorbed oxygen, and then the remaining holes are formed, so the resistance of the PcCo/rGO complex is lowered and the sensor is recovered.^30,46^ Further, twelve dynamic response and recovery cycles were tested exposing to the NH_3_ concentration from 100 ppb to 200 ppm (Fig. 5B). Clearly, the cpoPcCo/rGO sensor exhibited a stable and reversible response signal towards NH_3_, even at a low concentration of NH_3_ (100 ppb). Moreover, it can be observed that the response gradually increased as the target gas concentration increased from 100 ppb to 200 ppm. The response of the cpoPcCo/rGO sensor with the concentration of NH_3_ exhibits two good linear responses, as shown in Fig. 5C, 3.63% per ppm NH_3_ for concentration ranging from 0.1–6 ppm, and 0.14% per ppm NH_3_ for concentration ranging from 12–100 ppm. A linear regression equation of *S* = 3.63*C* (ppm) + 2.23 (*R*^2^ = 0.998) was derived from the calibration curve. Reproducibility and stability are very important factors in evaluating gas sensors. The cycling stability and long-term stability towards 100 ppm NH_3_ were measured at the room temperature. After 10 cycles, the cpoPcCo/rGO sensor still maintained a fast response and recovery capability, and the response showed no difference, as shown in Fig. 5D. In addition, the response changes only 8.0% during sixty days, which also shows that the cpoPcCo/rGO sensor has excellent long-term stability towards NH_3_. The NH_3_ sensing performance of other three RPcCo/rGO sensors were conducted and are shown in Fig. S10–S12,† which also show high sensitivity, good linear response, long-term stability and reproducibility, which are also significantly superior to the previously reported sensors (as shown in Table S1†). Moreover, in order to investigate the effect of cobalt phthalocyanine content on the sensing performances based on the rGO-based hybrids, the cpoPcCo/rGO2 and cpoPcCo/rGO3 were prepared by sonicating GO (0.100 g) DMF solution with cpoPcCo (0.100 g) and cpoPcCo (0.300 g) DMF solution. The response of cpoPcCo/rGO2 and cpoPcCo/rGO3 sensors upon varying the concentration of NH_3_ was measured. Fig. S13† shows that the response of the cpoPcCo/rGO2 and cpoPcCo/rGO3 sensors increase as the concentration of NH_3_ increases, which is consistent with the characteristics of the cpoPcCo/rGO sensor. Moreover, it is clear that the cpoPcCo/rGO sensor exhibits the highest response (42.5% to 100 ppm NH_3_).

### 3.3 Gas sensitivity mechanism

The reasons for the excellent gas sensing performance of RPcCo/rGO sensors can be ascribed to the synergistic effects of RPcCo and rGO. The schematic of the RPcCo/rGO hybrid gas-sensing mechanism for NH_3_ is exhibited in Fig. S14a.† First, when RPcCo/rGO sensors are exposed to ambient air, the oxygen molecules can rapidly and spontaneously adsorb on the center metal atoms of RPcCo, and form adsorbed oxygen species (O_2_^−^) by further capturing electrons from the conduction band of RPcCo.^39,47,48^ When the RPcCo/rGO sensors were exposed to reducing gases (for instance, NH_3_), these gas molecules reacted with the adsorbed oxygen species, as indicated in the following equation: 4NH_3_ (g) + 3O_2_^−^(ads) → 2N_2_ (g) + 6H_2_O (g) + 6e^−^.^39^ This process releases the electrons, and then rapidly transfer to the rGOs as the electron transfer energy barrier between RPcCo and rGO nanomaterial is low. Fig. S14b–e† show the optical absorption spectra of cpoPcCo and rGO, from which the band gaps of cpoPcCo and rGO can be estimated from the plot of (*Ahν*)^2^ (for direct band gap) *versus* photon energy (*hν*). The intercepts of the tangent to the *x*-axis give a good approximation of the band gap of cpoPcCo and rGO to be 1.83 and 1.13 eV, respectively. It indicates that cpoPcCo has lower conductivity compared to highly conductive rGO. Moreover, cpoPcCo strongly interacts with rGO *via* π–π interactions. Thus, more electrons transfer from cpoPcCo to rGO due to the higher surface area of the cpoPcCo/rGO hybrids. Finally, the holes in the P-type rGOs recombined with electrons, which lead to an increase in resistance. Contrary to the response process, the RPcCo/rGO hybrids can lose electrons quickly by reacting with the reappearing oxygen molecules in the desorption process of ammonia gas, which promotes the sensor resistance to rapidly return to the baseline position. The response of four RPcCo/rGO sensors increases as the concentration of NH_3_ increases, which is the characteristic of P-type semiconductors. To verify the mechanism, the signal resistors of the RPcCo/rGO sensors were investigated when it is exposed to strong oxidants, *e.g.*, NO_2_ (Fig. S15†). Contrary to NH_3_, the resistance reduces sharply when RPcCo/rGO sensors are exposed to NO_2_. NO_2_ can trap the electrons from RPcCo/rGO hybrids and obtain more holes in hybrids, leading to the reduced resistance (see Fig. S15†), which is consistent with the charge-transfer mechanism. Moreover, the response of the cpoPcCo/rGO sensor to 10 ppm NO_2_ is about 1.5% which is far less than the response of the cpoPcCo/rGO sensor to NH_3_, and the recovery characteristic is also poor. The result indicates that the cpoPcCo/rGO hybrid has an excellent selectivity toward NH_3_.

To verify the above-mentioned mechanism, the cpoPcCo-rGO and cmpoPcCo-rGO hybrids were successfully synthesized based on covalent bonding, as characterized by its UV-vis, FTIR spectra and TG analysis (Fig. S16 and S17†). However, the NH_3_-sensing response of the cpoPcCo-rGO and cmpoPcCo-rGO hybrids was less than that of the cpoPcCo/rGO and cmpoPcCo/rGO hybrids (Fig. S18†). These results indicate that the non-covalent strategy is more advantageous, which is mainly ascribed to the synergetic effect of RPcCo and rGO due to the large-surface-area structure for NH_3_ diffusion, the abundant active sites to adsorb NH_3_, and excellent conductivity for efficient electron transport, particularly the effect of RPcCo.

**Fig. 6:**
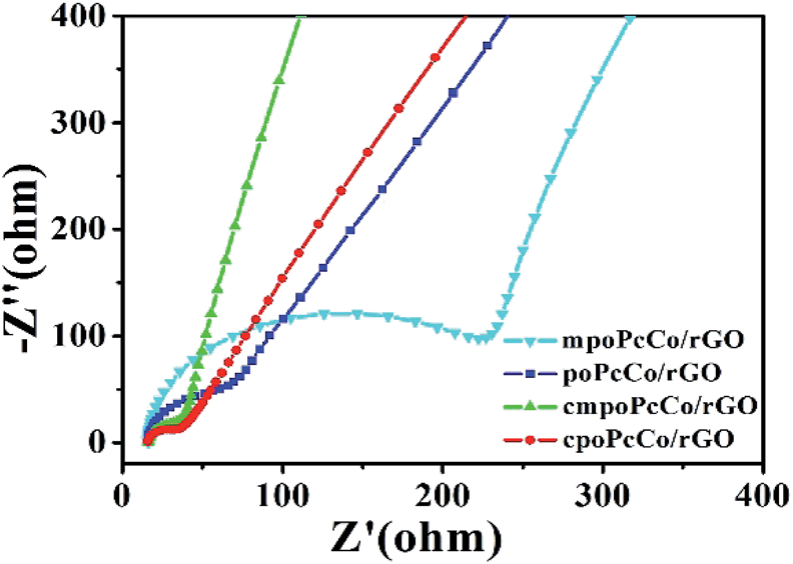
Nyquist plots of RPcCo/rGO hybrids.

The effect of different RPcCos on the NH_3_ sensing properties of RPcCo/rGO hybrids was further studied by the first-principles density functional theory (DFT). Considering fewer interactions of NH_3_ and rGO,^17^ only the interactions between NH_3_ with the RPcCos were investigated. The calculation results are shown in Table 1. The bond length of cpoPcCo/rGO (*r*_Co–N_, 2.193) was less than that of the other hybrids. The *r*_Co–N_ parameter illustrates the adsorption interactions between NH_3_ and RPcCos. A smaller bond length means stronger adsorption between NH_3_ and RPcCos. It can be further confirmed by calculating the adsorption energy between the RPcCo–NH_3_ systems, which can be seen to be negative, indicating that the NH_3_ adsorption on the RPcCo complex is an exothermic process, which will facilitate the formation of a stable structure.^43,49^ The adsorption energy of cpoPcCo–NH_3_ (−23.1 kcal mol^−1^) is more negative than that of the other hybrids, hence, NH_3_ shows affinity to interact on cpoPcCo/rGO. To elucidate the charge transfer between the NH_3_ and RPcCos, the NBO calculations were performed. In general, the net charges on NH_3_ are positive (Table 1), implying that there occurs electron transfer from NH_3_ to RPcCos. Charge transfer indicates a charge transfer sensing mechanism for the RPcCo/rGO sensors, which agrees well with the experimental observation. It is noted that there is much charge transfer in the adsorption of NH_3_ on cpoPcCo (0.189 eV) than that of other systems. In short, the NH_3_ sensitivity of cpoPcCo/rGO hybrid is the highest. Moreover, the cmpoPcCo/rGO hybrid shows also a better response than other two kinds of hybrids, further illustrating that the carboxyl substituent played a critical role in gas sensitivity. To further verify the charge transfer between RPcCos and NH_3_, the electrochemical impedance spectroscopy (EIS) of RPcCo/rGO hybrids was performed (Fig. 6). Fig. S19† shows the EIS of rGO. The fitting EIS parameters of the rGO and RPcCo/rGO hybrids were analyzed in Table 2. The *R*_b_ and *R*_ct_ of the cpoPcCo/rGO (16.61, 18.74 Ω) and cmpoPcCo/rGO (16.92, 27.58 Ω) hybrids are obviously lower than those of poPcCo/rGO (18.30, 234.15 Ω), mpoPcCo/rGO (19.03, 381.32 Ω) and rGO (28.53, 785.80 Ω), respectively. *R*_b_ is the uncompensated resistance of the electrolyte, separator and electrode. *R*_ct_ is related to the electrical conductivity and electron transport of the material. The lower the values of *R*_b_ and *R*_ct_, the stronger the electron transport capability. Therefore, the cpoPcCo/rGO and cmpoPcCo/rGO hybrids exhibit a superior response to NH_3_ over the other hybrids, which is consistent with the DFT analysis.

**Table 1:** Calculated adsorption energies, major bond distance, and net charges in the adsorption of NH_3_ on the RPcCos

Sub.^a^	Net charge^b^	*d*(Co–N)^c^ (Å)	Adsorption energy (kcal mol^−1^)
cpoPcCo	0.189	2.193	−23.1
cmpoPcCo	0.187	2.194	−22.4
poPcCo	0.186	2.195	−22.0
mpoPcCo	0.184	2.197	−21.7

a The substrate.

b The net charge on NH_3_.

c Distance between cobalt and NH_3_.

**Table 2:** Fitted impedance parameters of rGO and PcCo/rGO electrode

Samples	*R*_b_ (Ω)	*R*_ct_ (Ω)
cpoPcCo/rGO	16.61	18.74
cmpoPcCo/rGO	16.92	27.58
poPcCo/rGO	18.30	234.15
mpoPcCo/rGO	19.03	381.32
rGO	28.53	785.80

## 4. Conclusions

In summary, RPcCo/rGO hybrids were successfully prepared by coating four different phenoxyl substituents PcCos on the surface of rGO *via* non-covalent interactions, which is advantageous for maintaining the structural superiority of rGOs as well as the active sites for the NH_3_ adsorption by PcCos. Due to the large-surface-area structure for NH_3_ diffusion, the abundant active sites to adsorb NH_3_, and excellent conductivity for efficient electron transport, the obtained sensors exhibited excellent sensitivity, selectivity, good reproducibility and perfect response–concentration linearity to NH_3_ at room temperature. In particular, the cpoPcCo/rGO based sensor shows a high and fast response for a low concentration of NH_3_ (∼2.5 and 45 s for 100 ppb of NH_3_), a ppb level detection and superior stability over 60 days. Besides, the sensitivity order of PcCo/rGO hybrids to NH_3_ is cpoPcCo/rGO > cmpoPcCo/rGO > poPcCo/rGO > mpoPcCo/rGO, and is supported by the first-principles density functional theory and impedance. Therefore, it is a valid pathway to design the functionalized rGO-based gas-sensing materials by regulating the substituent groups of RPcCo.

## Conflicts of interest

There are no conflicts to declare.

## Supplementary Material

RA-009-C9RA08065A-s001

## References

[cit1] Fei H. F., Wu G., Cheng W. Y., Yan W. J., Xu H. J., Zhang D., Zhao Y. F., Lv Y. H., Chen Y. H., Zhang L., Coileain C. Ó., Heng C. L., Chang C. R., Wu H. C. (2019). ACS Omega.

[cit2] Biswas M. R. U. D., Oh W. C. (2019). RSC Adv..

[cit3] Li S. Q., Diao Y. J., Yang Z. J., He J. M., Wang J., Liu C. C., Liu F. M., Lu H. Y., Yan X., Sun P., Lu G. Y. (2018). Sens. Actuators, B.

[cit4] Acharya L. S. K., Kumara A., Barika B., Nayaka P. S., Tripathyb N., Karb J. P., Dasha P. (2018). Sens. Actuators, B.

[cit5] Ma J., Guo X. Y., Ying Y. P., Liu D. H., Zhong C. L. (2017). Chem. Eng. J..

[cit6] Ramezanzadeh B., Haeri Z., Ramezanzadeh M. (2016). Chem. Eng. J..

[cit7] Novoselov K. S., Geim A. K., Morozov S. V., Jiang D., Zhang Y., Dubonos S. V., Grigorieva I. V., Firsov A. A. (2004). Science.

[cit8] Schedin F., Geim A. K., Morozov S. V., Hill E. W., Blake P., Katsnelson M. I., Novoselov K. S. (2007). Nat. Mater..

[cit9] Wehling T. O., Novoselov K. S., Morozov S. V., Vdovin E. E., Katsnelson M. I., Geim A. K., Lichtenstein A. I. (2008). Nano Lett..

[cit10] Leenaerts O., Partoens B., Peeters F. M. (2008). Phys. Rev. B: Condens. Matter Mater. Phys..

[cit11] Chen G., Paronyan T. M., Harutyunyan A. R. (2012). Appl. Phys. Lett..

[cit12] Li W. W., Li X., Cai L., Sun Y. L., Sun M. X., Xie D. (2018). J. Nanosci. Nanotechnol..

[cit13] Zhou Y., Lin X. G., Huang Y. K., Guo Y. C., Gao C., Xie G. Z., Jiang Y. D. (2016). Sens. Actuators, B.

[cit14] Lu G. H., Park S. J., Yu K. H., Ruoff R. S., Ocola L. E., Rosenmann D., Chen J. H. (2011). ACS Nano.

[cit15] Allen M. J., Tung V. C., Kaner R. B. (2010). Chem. Rev..

[cit16] Mao S., Cui S. M., Lu G. H., Yu K. H., Wen Z. H., Chen J. H. (2012). J. Mater. Chem..

[cit17] Yuan W. J., Shi G. Q. (2013). J. Mater. Chem. A.

[cit18] Hakimi M., Salehi A., Boroumand F. A., Mosleh N. (2018). IEEE Sens. J..

[cit19] Karaduman I., Er E., Celikkan H., Erk N., Acar S. (2017). J. Alloys Compd..

[cit20] Ye Z. B., Tai H. L., Guo R., Yuan Z., Liu C. H., Su Y. J., Chen Z., Jiang Y. D. (2017). Appl. Surf. Sci..

[cit21] Sun Q. Q., Feng W. G., Yang P., You G. Q., Chen Y. L. (2018). New J. Chem..

[cit22] Linstead R. P. (1934). J. Chem. Soc..

[cit23] Sizun T., Bouvet M., Suisse J. M. (2012). Talanta.

[cit24] Dent C. E., Linstead R. P., Lowe A. R. (1934). J. Chem. Soc..

[cit25] Ma H., Zhang H. X., Tong M. Q., Cao J. D., Wu W. (2019). RSC Adv..

[cit26] Zhang J., Liu X. H., Neri G., Pinna N. (2016). Adv. Mater..

[cit27] Huang X. L., Hu N. T., Gao R. G., Yu Y., Wang Y. Y., Yang Z., Kong E. S. W., Wei H., Zhang Y. F. (2012). J. Mater. Chem..

[cit28] Wang B., Wang X. L., Li X. C., Guo Z. J., Zhou X., Wu Y. Q. (2018). RSC Adv..

[cit29] Zhou X. Q., Wang X. L., Wang B., Chen Z. M., He C. Y., Wu Y. Q. (2014). Sens. Actuators, B.

[cit30] Wu H., Chen Z. M., Zhang J. L., Wu F., He C. Y., Wang B., Wu Y. Q., Ren Z. Y. (2016). J. Mater. Chem. A.

[cit31] Wang B., Zhou X. Q., Wu Y. Q., Chen Z. M., He C. Y. (2012). Sens. Actuators, B.

[cit32] Yanaia T., Tew D. P., Handyb N. C. (2004). Chem. Phys. Lett..

[cit33] Glendening E. D. , Carpenter A. E. and Weinhold F., NBO, version 3.1, 1995

[cit34] Frisch M. J. , et al., Gaussian 09, Revision B01, Gaussian, Inc., Wallingford, CT, 2009

[cit35] Günsel A., Bilgiçli A. T., Pişkin H., Delibaş N. Ç., Yarasir M. N., Gündüz B. (2018). New J. Chem..

[cit36] Zhao H. Z., Zhang Y., Zhao B., Chang Y. Y., Li Z. S. (2012). Environ. Sci. Technol..

[cit37] Souza F. D., Ito O. (2009). Chem. Commun..

[cit38] Harbeck S., Emirik O. F., Gurol I., Gurek A. G., Ozturk Z. Z., Ahsen V. (2013). Sens. Actuators, B.

[cit39] Wu H., Chen Z. M., Zhang J. L., Wu F., He C. H., Ren Z. Y., Wu Y. Q. (2017). Chem. Mater..

[cit40] Chunder A., Pal T., Khondaker S. I., Zhai L. (2010). J. Phys. Chem. C.

[cit41] Zhu J. H., Li Y. X., Chen Y., Wang J., Zhang B., Zhang J. J. (2011). Carbon.

[cit42] Huang X. L., Hu N. T., Gao R. G., Yu Y., Wang Y. Y., Yang Z., Kong E. S. W., Weia H., Zhang Y. F. (2012). J. Mater. Chem..

[cit43] Li Y., Wang B., Yu Z. Y., Zhou X. Q., Kang D., Wu Y. Q., Chen Z. M., He C. Y., Zhou X. (2017). RSC Adv..

[cit44] Mao S., Lu G. H., Chen J. H. (2014). J. Mater. Chem. A.

[cit45] Bohrer F. I., Colesniuc C. N., Park J., Ruidiaz M. E., Schuller I. K., Kummel A. C., Trogler W. C. (2009). J. Am. Chem. Soc..

[cit46] Manouchehrian M., Larijani M. M., Elahi S. M. (2015). Mater. Res. Bull..

[cit47] Liu H., Li M., Voznyy O., Hu L., Fu Q. Y., Zhou D. X., Xia Z., Sargent E. H., Tang J. (2014). Adv. Mater..

[cit48] Liu Y. L., Wang H. R., Chen K. Q., Yang T. Q., Yang S., Chen W. (2019). ACS Appl. Mater. Interfaces.

[cit49] Cao Z. F., Chen Q. B., Lu Y. X., Liu H. L., Hu Y. (2013). Int. J. Quantum Chem..

