# Supplementary material for: A high-sensitive room temperature gas sensor based on cobalt phthalocyanines and reduced graphene oxide nanohybrids for the ppb-levels of ammonia detection
Source: RSC Adv. 2019 Nov 18;9(64):37518–25. doi: 10.1039/c9ra08065a (PMC9075529; doi:10.1039/c9ra08065a)
Supplement: RA-009-C9RA08065A-s001 [file RA-009-C9RA08065A-s001.pdf]

Electronic Supplementary Material (ESI) for RSC Advances.
This journal is © The Royal Society of Chemistry 2019

Highsensitive room temperature gas sensor based on cobalt phthalocyanines and reduced graphene oxide nanohybrids for ppb-levels of ammonia detection†

ZhiJiang Guo,[a] Bin Wang,*[a] Xiaolin Wang,[b] Yong Li,[a] Shijie Gai,[a] Yiqun Wu,[ac] and XiaoLi Cheng,[a]

[a] Key Laboratory of Functional Inorganic Material Chemistr, Ministry of Education, School of Chemistry and Materials Science, Heilongjiang University, Harbin 150080, P. R. China. E-mail: wangbin@hlju.edu.cn.

[b] School of Material and Chemical Engineering, Heilongjiang Institute of Technology, Harbin 150050, P. R. China

[c] Shanghai Institute of Optics and Fine Mechanics, Chinese Academy of Sciences, Shanghai 201800, P. R. China.

Tel.: +86 451 86609121

Fax: +86 451 86673647

1. Experimental detail

1.1 Materials

All chemicals were analytical grade and commercially available and used without further purification. 3-nitrophthalonitrile was purchased from Sigma-Aldrich Co . LLC., and was used without further purification. The synthesis scheme of tetra-β-carboxylphenoxylphthalocyanine cobalt (cpoPcCo), tetra-β-(4-carboxy-3-methoxyphenoxy)phthalocyanine cobalt (cmpoPcCo), tetra-β-phenoxylphthalocyanine cobalt (poPcCo), and tetra-β-(3-methoxyphenoxy)phthalocyanine cobalt (mpoPcCo) is shown in scheme S1.

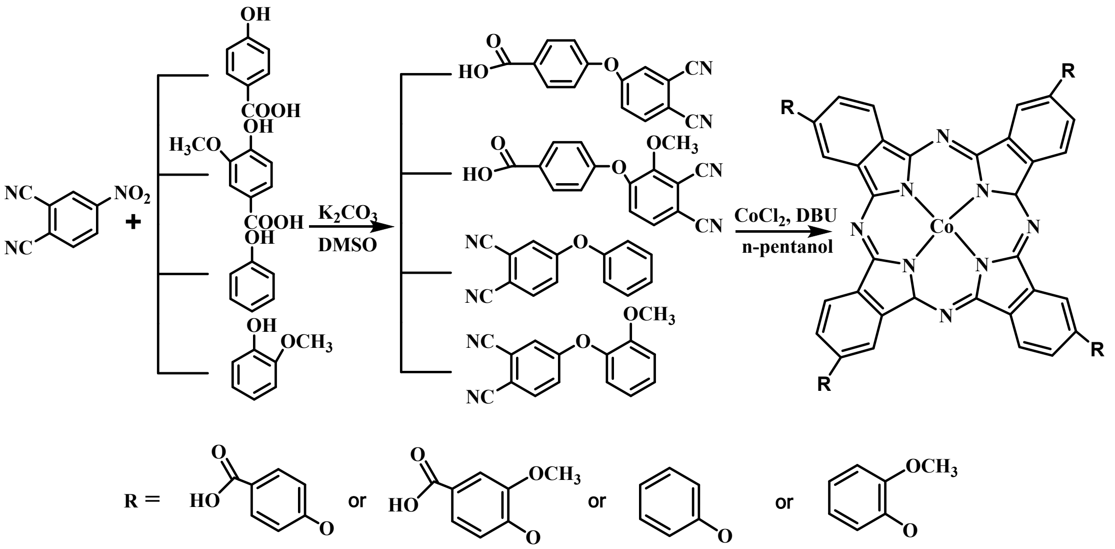

Scheme S1. Synthesis scheme of cpoPcCo, cmpoPcCo, poPcCo and mcpoPcCo.

1.2 The structure for gas sensors

Al_2O_3 ceramic substrate is used for gas sensors. TaN, TiW, Ni and Au are sputtered on substrate as resistive layer, supporting layer, solder mask and conductor layer, respectively. Standard photolithography is then used to create Au interdigitated electrodes with 180 μm electrode widths, and 50 μm electrode gaps.

1.3 Characterization

EI and MALDI-TOF mass spectra were performed using an Agilent spectrometer (HP

5973N) and a Bruker microflex LT (Bruker Daltonics, Bremen, Germany) mass spectrometer, respectively. Scanning electron microscopy (SEM) images were recorded with a Hitachi S-4800 field emission scanning electron microscope operating at 15 kV. Samples were drop-deposited onto the interdigitated electrodes and measured directly. UV/Vis absorption spectra were recorded with an UV-2700 spectrometer (SHIMADZU, Jap). FT-IR spectra were recorded on a Spectrum two spectrometer (PerkinElmer).

1.4 Synthesis of 4-(4-carboxyphenoxy)phthalonitrile

4-Nitrophthalonitrile (6.93 g, 0.040 mol) was dissolved in DMSO (160 mL) under a nitrogen atmosphere, and then p-hydroxybenzoic acid (10.97 g, 0.080 mol) was added to the solution. After stirring for 30 minutes, anhydrous potassium carbonate (K_2CO_3) (19.35 g, 0.14 mol) was added portion wise over 3 days with constant stirring. The reaction mixture was stirred at room temperature for 5 days under a nitrogen atmosphere. The solid residue was removed by filtration, and the filtrate was acidified to pH 1-2 and then allowed to stand for 10 minutes. The resulting precipitate was collected by filtration, washed with distilled water and dried in vacuo. Recrystallization twice with methanol gave finally white 4-(4-carboxyphenoxy)phthalonitrile. Similarly, after the conversion of p-hydroxybenzoic acid to sodium 4-hydroxy-3-methoxybenzoate, phenol and guaiacol. The same method can be used to prepare 4-(4-carboxyl-3-methoxybenzonitrile) phthalonitrile, 4-phenoxyphthalonitrile and 4-(3-methoxyphenoxybenzonitrile) phthalonitrile.

1.5 Synthesis of tetra-β-carboxyphenoxyphthalocyanine cobalt

At room temperature, 4-(4-carboxyphenoxy) phthalonitrile (2.64 g, 0.010 MOL), anhydrous cobalt chloride (II) (0.39 g, 0.003 MOL) and DBU (4.0 mL) were added to distilled n-pentanol (60 mL). The subsequent mixture was continuously stirred under reflux for 20 hours under a nitrogen atmosphere. After naturally cooling to about 20 ℃, the precipitate was filtered, washed sequentially with methanol (50 mL) and

acetone (50 mL) and dissolved in potassium hydroxide solution (1 mol L^{-1}, 100 mL). The solid residue in the solution was removed by filtration, and the filtrate was acidified to pH 3-4 by adding concentrated hydrochloric acid with stirring, and left overnight. The resulting precipitate was collected by centrifugation, washed with distilled water until the pH of the supernatant was about 7, and then dried in a vacuum oven at 50 ° C to obtain a purple-black crystal cpoPcCo. MALDI-TOF-MS Calcd (found): m/z = 1115.02 (1117.16) (Fig. S1). Three other phthalocyanines can be synthesized by replacing 4-(4-carboxyphenoxy) phthalonitrile with 4-(4-carboxy-3-methoxyphenoxy) phthalonitrile, 4-phenoxy phthalonitrile and 4-(3-methoxyphenoxy) phthalonitrile. That is tetra-β-carboxymethoxyphenoxyphthalocyanine cobalt, MALDI-TOF-MS Calcd (found): m/z = 1239.21 (1237.21) [M^+].

tetra-β-phenoxyphthalocyanine cobalt, MALDI-TOF-MS Calcd (found): m/z = 942.19 (941.20) [M^+]. tetra-β-methoxyphenoxy phthalocyanine cobalt, MALDI-TOF-MS Calcd (found): m/z = 1062.22 (1061.25) [M^+].

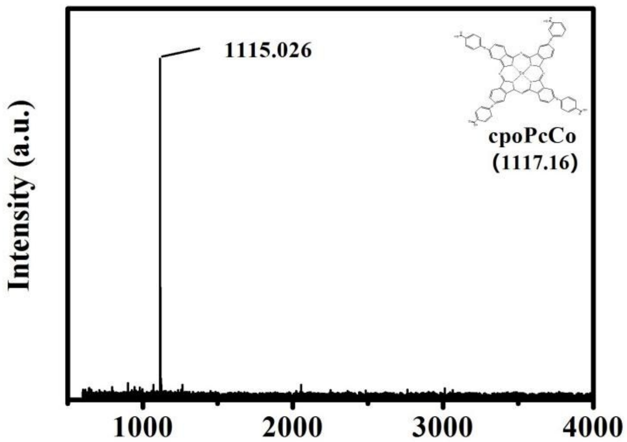

Fig. S1 MALDI-TOF-MS of cpoPcCo.

2. Result and discussion

Fig. S2. Uv-Vis spectra of rGO, RPcCo, and RPcCo/rGO hybrids.

Fig. S3. FT-IR spectra of rGO, RPcCo, and RPcCo/rGO hybrids.

Fig. S4. TG spectra of rGO, RPcCo, and RPcCo/rGO hybrids.

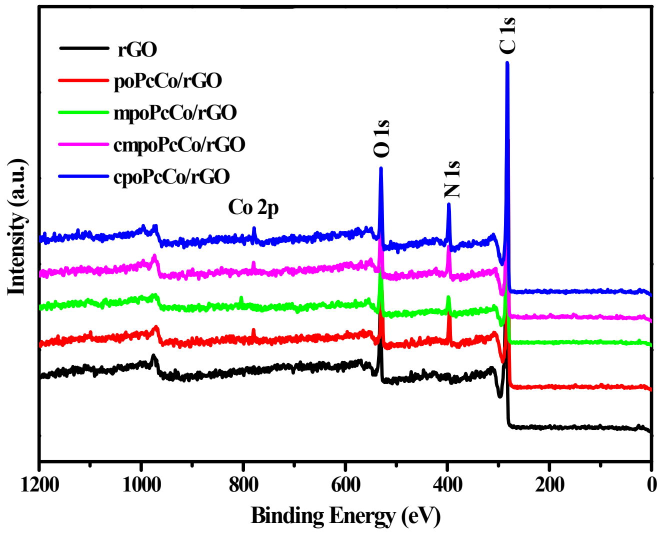

Fig. S5. XPS full survey of rGO and RPcCo/rGO hybrids.

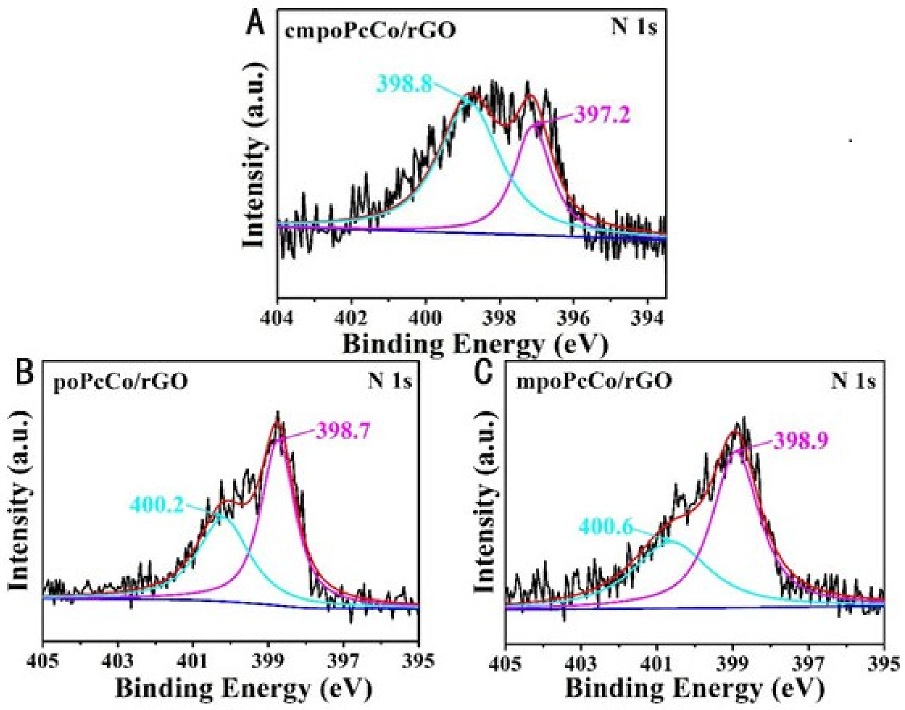

Fig. S6. N 1s XPS spectra of cmpoPcCo, poPcCo and mpoPcCo hybrids

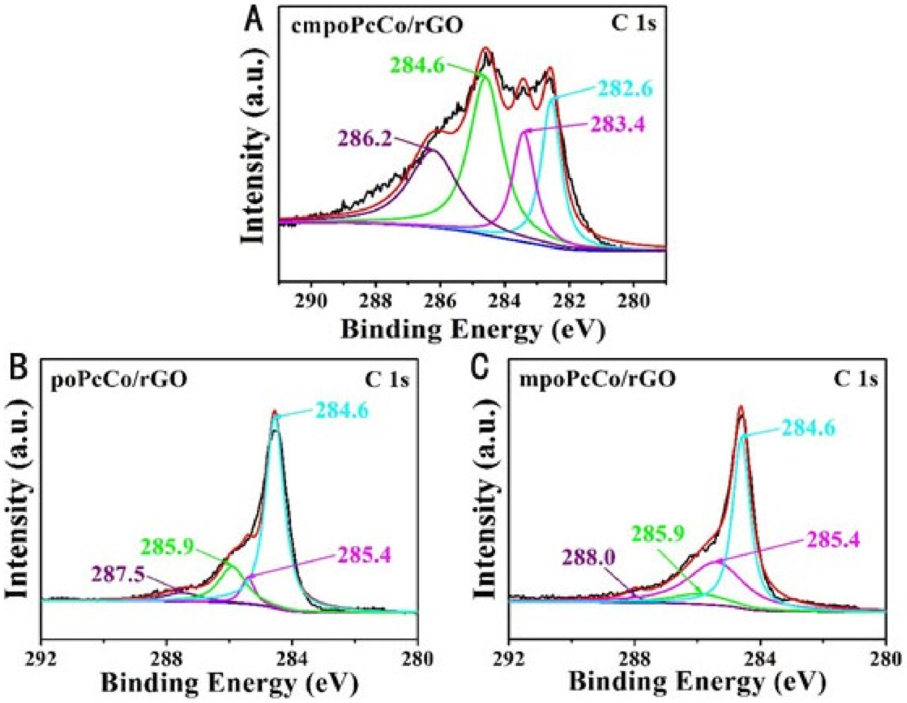

Fig. S7. C 1s XPS spectra of cmpoPcCo, poPcCo and mpoPcCo hybrids

Fig. S8 SEM images of RPcCo/rGO hybrids on the interdigital electrode.

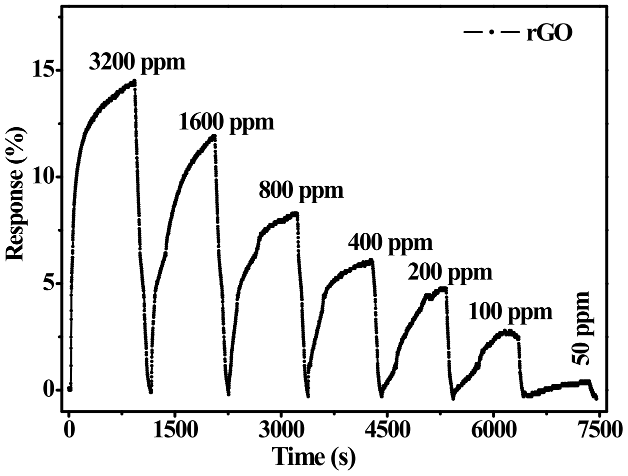

Fig. S9. Response recovery curve of rGO at room temperature.

Fig. S10. (A) Response of cmpoPcCo/rGO hybrid sensor exposure to 100 ppm NH_3 and recovery under UV light or without UV light; (B) resistance of cmpoPcCo/rGO hybrid sensor upon exposure to varying concentrations of NH_3; (C) relationship of the response of cmpoPcCo/rGO hybrid sensor to the concentration NH_3; (D) ten sensing cycles of cmpoPcCo/rGO hybrid sensor to 100 ppm NH_3 (inset: the reproducibility characteristics of the cmpoPcCo/rGO hybrid sensor to 100 ppm NH_3within 60 days) at 29 ℃.

Fig. S11. (A) Response of mpoPcCo/rGO hybrid sensor exposure to 100 ppm NH_3 and recovery under UV light or without UV light; (B) resistance of mpoPcCo/rGO hybrid sensor upon exposure to varying concentrations of NH_3; (C) relationship of the response of mpoPcCo/rGO hybrid sensor to the concentration NH_3; (D) ten sensing cycles of mpoPcCo/rGO hybrid sensor to 100 ppm NH_3 (inset: the reproducibility characteristics of the mpoPcCo/rGO hybrid sensor to 100 ppm NH_3within 60 days) at 29 ℃.

Fig. S12. (A) Response of poPcCo/rGO hybrid sensor exposure to 100 ppm NH_3 and recovery under UV light or without UV light; (B) resistance of poPcCo/rGO hybrid sensor upon exposure to varying concentrations of NH_3; (C) relationship of the response of poPcCo/rGO hybrid sensor to the concentration NH_3; (D) ten sensing cycles of poPcCo/rGO hybrid sensor to 100 ppm NH_3 (inset: the reproducibility characteristics of the poPcCo/rGO hybrid sensor to 100 ppm NH_3within 60 days) at 29 ℃.

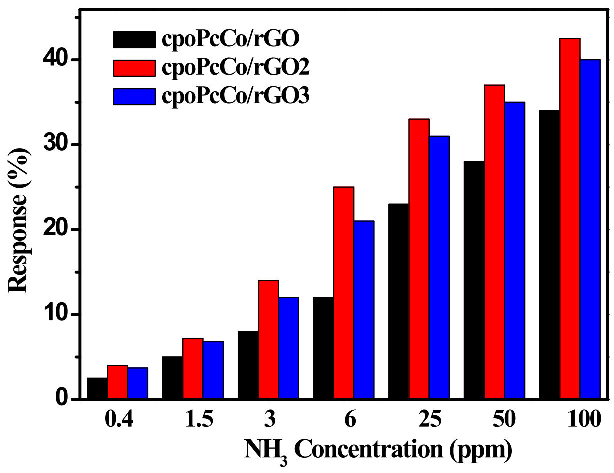

Fig. S13 Response of cpoPcCo/rGO, cpoPcCo/rGO2 and cpoPcCo/rGO3 sensors upon varying the concentration of NH_3.

Fig. S14. Schematic diagram of the NH_3-sensing mechanism of the sensor (a); UV-Vis absorption spectra (b,d) and band gap energies (c,e) of cpoPcCo and rGO.

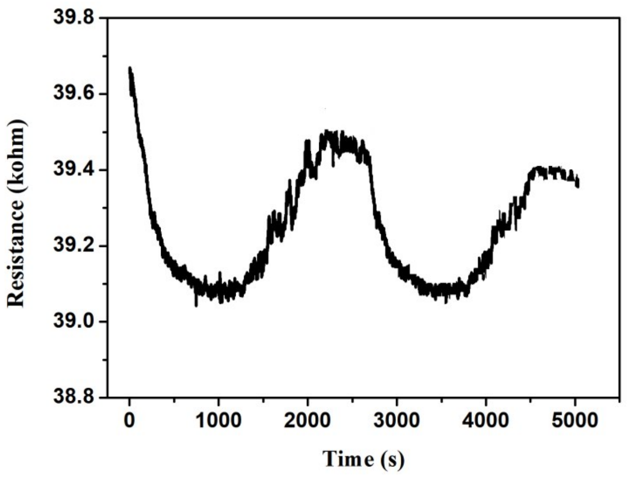

Fig. S15. Resistance of cpoPcCo/rGO hybrid sensor upon exposure to 10 ppm of NO_2 at 29 °C.

Fig. S16. (A) UV-vis spectra; (B) FT-IR spectra; (C) TG profiles of rGO, cpoPcCo and cpoPcCo-rGO hybrid.

A

Fig. S17. (A) UV-vis spectra; (B) FT-IR spectra; (C) TG profiles of rGO, cmpoPcCo and cmpoPcCo-rGO hybrid.

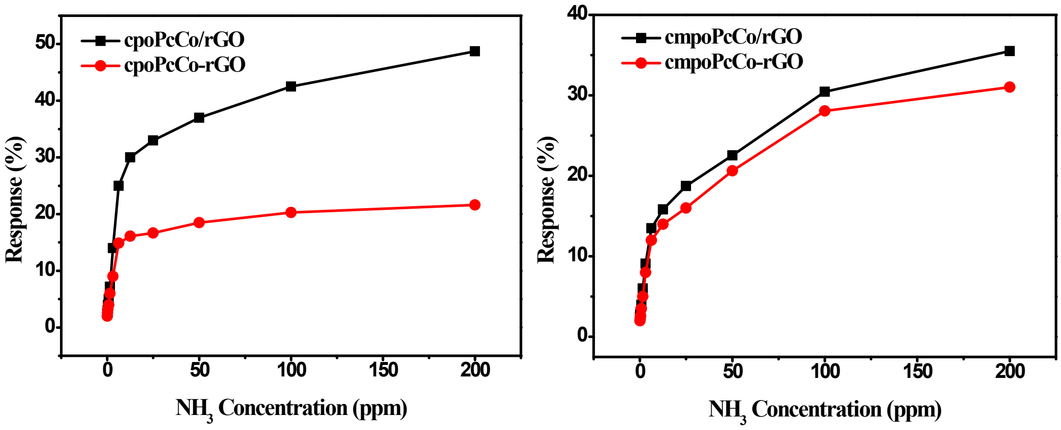

Fig. S18. Relationship of the response of RPcCo/rGO and RPcCo-rGO hybrid sensors to the concentration of NH_3.

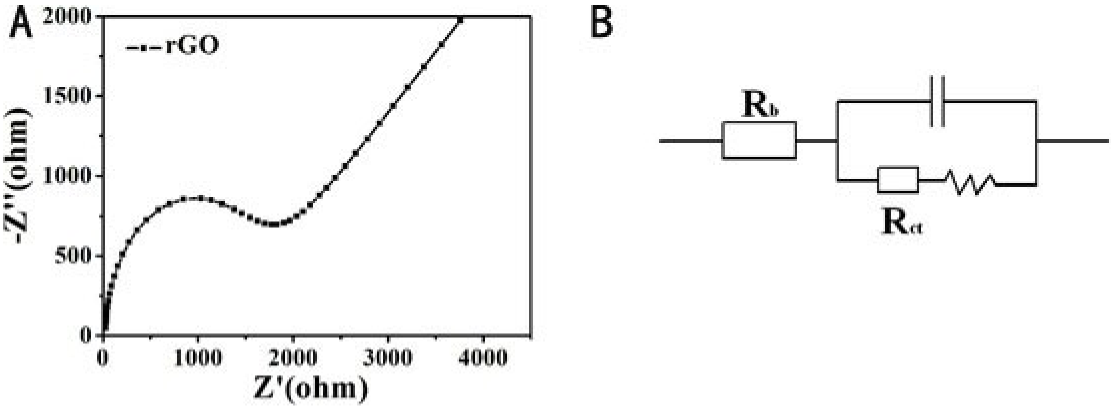

Fig. S19. Nyquist plots of rGO and Equivalent circuit diagram.

Table S1. Comparison of the detection performances of different NH_3 sensors

Sensor material	Response(%)/ Detection conc. (ppm) [b]	Detection limit(ppm)[a]	Working temperature (°C)[c]	Response time(s)/Detection conc.(ppm)[b]	Recovery time (s)/Detection conc.(ppm)[b]	Detection range (ppm)	Ref.
CuPc/N-graphene/PEDOT-PSS	8/50	1	RT	138/200	63/200	1-1000	1
PQT-12	8.6/100	0.3	RT	8/100	103/100	10-100	2
PANI-CeO_2	262.7/50	0.016	RT	600/50	1800/50	10-50	3
coral-shaped Dy_2O_3	7.754/50	0.1	RT	17.4/50	840/50	0.1-100	4
RGO/MnO_2 + PANI	25.1/5	5	RT	1080/5	240/5	5-50	5
NiV_2O_6	92/100	20	120	9/100	16/100	20-100	6
SnO_2/SnS_2	2.48/100	10	RT	21/100	110/100	10-500	7
In_2O_3/PANI	52.4/1000	100	RT	500/1000	500/1000	100-1000	8
Flower-shaped SnS_2	7.4/100	0.5	200	50.6/100	624/100	0.5-100	9
2D SnS_2	4.2/500	20	RT	16/500	450/500	20-800	10
Pt/Cr/ZnO	81.6/1000	10	300	74/1000	29/1000	10-1000	11
Co_3O_4	11.2/100	10	160	2/100	10/100	10-200	12
TeO_2	58/500	100	170	186/500	336/500	100-500	13
ZnO/RGO	19.2/50	0.05	RT	50/50	250/50	0.05-5000	14

$[Au^{III}Cl(L)]$ (PF6)	27.5/80	1	RT	8/80	11/80	1-200	15
RGO	5.7/50	5	RT	24/50	780/50	5-50	16
$Pd/SnO_2/$ RGO	19.6/100	5	RT	420/100	3000/100	5-300	17
Sr/SnO_2	54.23/2000	10	RT	6/2000	—	10-2000	18
SnO_2/rGO	30/200	500	RT	8/200	13/200	500-3000	19
MWCNTs	18/500	100	RT	798/500	464/500	100,200,500	20
Ag_3PO_4	52/100	10	50	276/100	1338/100	10-1000	21
cpoPcCo/rGO	2.5/0.1<br>42.4/100	3.7 ppb	RT	450/0.1<br>450/100	30/0.1<br>120/100	0.1-200	This work[d]
cmpoPcCo/rGO	2.5/0.1<br>31.4/100	16 ppb	RT	540/0.1<br>540/100	30/0.1<br>120/100	0.1-200	
poPcCo/rGO	2.0/0.1<br>27.3/100	21 ppb	RT	450/0.1<br>420/100	36/0.1<br>78/100	0.1-200	
mpoPcCo/rGO	1.5/0.1<br>22.1/100	23 ppb	RT	480/0.1<br>480/100	84/0.1<br>126/100	0.1-200	

[a] If the sensor detection limit was not explicitly provided in the original report, then the lowest tested analyte concentration is listed.

[b] If the response (%), response time (s) or recovery time (s) of the sensor was not explicitly provided in the original report, then the estimate from the curve in that report is listed.

[c] RT, abbreviation for room temperature.

[d] sensor prepared with the RPcCo/rGO aqueous dispersion concentrations of 1.0 mg ml^{-1}

References

1. H.S. Dehsari, J.N. Gavgani, A. Hasani, M. Mahyari, E.K. shalamzari, A. Salehi, F.A. Taromi, Copper (II) phthalocyanine supported on three-dimensional nitrogen-doped graphene/PEDOT-PSS nanocomposite as highly selective and sensitive sensor for ammonia detection at room temperature, RSC Adv.,5 (2015) 79729-79737.

2. C. Kumar, G. Rawat, H. Kumar, Y. Kumar, R. Prakash, S. Jit, Flexible poly (3, 3 '''-dialkylquaterthiophene) based interdigitated metal-semiconductor-metal ammonia gas sensor, Sens. Actuators B, 255 (2018) 203-209.

3. C.H. Liu, H.L. Tai, P. Zhang, Z. Yuan, X.S. Du, G.Z. Xie,Y.D. Jiang.A high-performance flexible gas sensor based on self-assembled PANI-CeO_2 nanocomposite thin film for trace-level NH3 detection at room temperature. Sens. Actuators B, 261 (2018) 587–597.

4. X. Dong, X.L. Cheng, X.F. Zhang, L.L. Sui, Y.M. Xu, S. Gao, H. Zhao, L.H. Huo, A novel coral-shaped Dy_2O_3 gas sensor for high sensitivity NH_3 detection at room temperature.Sens. Actuators B 255 (2018) 1308–1315.

5. X. Huang, N. Hu, R. Gao, Y. Yu, Y. Wang, Z. Yang, E. Siu-Wai Kong, H. Wei, Y. Zhang, Reduced graphene oxide-polyaniline hybrid: Preparation, characterization and its applications for ammonia gas sensing. J. Mater. Chem. 22 (2012), 22488 .

6. C. Balamurugan, D.W. Lee, A selective NH_3 gas sensor based on mesoporous p-type NiV_2O_6 semiconducting nanorods synthesized using solution method. Sens. Actuators B, 192 (2014) 414–422.

7. R. Li, K. Jiang, S. Chen, Z. Lou, T.T. Huang, D. Chen, G.Z. Shen, SnO_2/SnS_2 nanotubes for flexible room temperature NH_3 gas sensors, RSC Adv., 2017, 7, 52503.

8. Z.Y. Pang, Q.X. Nie, A.F. Wei, J. Yang, F.L. Huang, F. Wei, J. Mater. Sci., 2017, 52, 686.

9. Y. Xiong, W. Xu, D. Ding, W. Lu, L. Zhu, Z. Zhu, Y. Wang, Q. Xue, Ultra-sensitive NH_3

sensor based on flower-shaped SnS_2 nanostructures with sub-ppm detection ability, J. Hazard Mater. 341 (2018) 159-167.

10. Z. Qin, K. Xu, H. Yue, H. Wang, J. Zhang, C. Ouyang, C. Xie, D. Zeng, Enhanced room-temperature NH_3 gas sensing by 2D SnS_2 with sulfur vacancies synthesized by chemical exfoliation, Sens. Actuators B, 262 (2018) 771-779.

11. T.Y. Chen, H.I. Chen, C.S. Hsu, C.C. Huang, J.S. Wu, P.C. Chou, W.C. Liu, Characteristics of ZnO nanorods-based ammonia gas sensors with a cross-linked configuration, Sens. Actuators B, 221 (2015) 491-498.

12. J.N. Deng, R. Zhang, L.L. Wang, Z. Lou, T. Zhang, Enhanced sensing performance of the Co_3O_4 hierarchical nanorods to NH_3 gas, Sens. Actuators B, 209 (2015) 449–455.

13. T. Siciliano, M.D. Giulio, M. Tepore, E. Filippo, G. Micocci, A. Tepore, Ammonia sensitivity of rf sputtered tellurium oxide thin films, Sens. Actuators B, 138 (2009) 550–555.

14. T. Wang, Z. Sun, D. Huang, Z. Yang, Q. Ji, N.T. Hu, G.L. Yin, D.N. He, H. Wei, Y.F. Zhang, Studies on NH_3 gas sensing by zinc oxide nanowire-reduced graphene oxide nanocomposites, Sens. Actuators B, 252 (2017) 284–294.

15. L. Tabrizi, H. Chiniforoshan, High-performance room temperature gas sensor based on gold(III) pincer complex with high sensitivity for NH_3, Sens. Actuators B, 245 (2017) 815–820.

16. W.W. Li, X. Li, L. Cai, Y.L. Sun, M.X. Sun, D. Xie, Reduced graphene oxide for room temperature ammonia (NH_3) gas sensor, J. Nanosci. Nanotechnol. 18 (2018) 7927–7932.

17. P.G. Su, L.Y. Yang, NH_3 gas sensor based on Pd/SnO_2/RGO ternary composite operated at room-temperature, Sens. Actuators B, 223 (2016) 202–208.

18. S. Xu, K. Kan, Y. Yang, C. Jiang, J. Gao, L.Q. Jing, P.K. Shen, L. Li, K.Y. Shi, Enhanced NH_3 gas sensing performance based on electrospun alkaline-earth metals composited SnO_2 nanofibers, J. Alloys Compd. 618 (2015) 240–247.

19. Y. Chen, W. Zhang, Q.S. Wu, A highly sensitive room-temperature sensing material for NH_3: SnO_2-nanorods coupled by rGO, Sens. Actuators B, 242 (2017) 1216–1226.

20. Z.Y. Gao, Z. Lou, S. Chen, L. Li, K. Jiang, Z.L. Fu, W. Han, G.Z. Shen, Fiber gas sensor-integrated smart face mask for room-temperature distinguishing of target gases, Nano Res. 11 (2017) 511–519.

21. F.F. Yan, G.W. Shen, X. Yang, T.J. Qi, J. Sun, X.H. Li, M.T. Zhang, Low operating temperature and highly selective NH_3 chemiresistive gas sensors based on Ag_3PO_4 semiconductor, Appl. Surf. Sci. 479 (2019) 1141-1147.
